# Nitrate Intake Promotes Shift in Muscle Fiber Type Composition during Sprint Interval Training in Hypoxia

**DOI:** 10.3389/fphys.2016.00233

**Published:** 2016-06-14

**Authors:** Stefan De Smet, Ruud Van Thienen, Louise Deldicque, Ruth James, Craig Sale, David J. Bishop, Peter Hespel

**Affiliations:** ^1^Exercise Physiology Research Group, Department of Kinesiology, Katholieke Universiteit LeuvenLeuven, Belgium; ^2^Institute of Neuroscience, Université Catholique de LouvainLouvain-la-Neuve, Belgium; ^3^Musculoskeletal Physiology Research Group, Sport, Health and Performance Enhancement Research Centre, School of Science and Technology, Nottingham Trent UniversityNottingham, UK; ^4^Institute of Sport, Exercise and Active Living, Victoria UniversityMelbourne, VIC, Australia; ^5^Department of Kinesiology, Bakala Academy–Athletic Performance Center, KU LeuvenLeuven, Belgium

**Keywords:** sprint interval training, hypoxia, intermittent hypoxic training, nitrate, muscle fiber type composition, muscle buffering capacity, carnosine, citrate synthase

## Abstract

**Purpose:** We investigated the effect of sprint interval training (SIT) in normoxia, vs. SIT in hypoxia alone or in conjunction with oral nitrate intake, on buffering capacity of homogenized muscle (βhm) and fiber type distribution, as well as on sprint and endurance performance.

**Methods:** Twenty-seven moderately-trained participants were allocated to one of three experimental groups: SIT in normoxia (20.9% F_i_O_2_) + placebo (N), SIT in hypoxia (15% F_i_O_2_) + placebo (H), or SIT in hypoxia + nitrate supplementation (HN). All participated in 5 weeks of SIT on a cycle ergometer (30-s sprints interspersed by 4.5 min recovery-intervals, 3 weekly sessions, 4–6 sprints per session). Nitrate (6.45 mmol NaNO_3_) or placebo capsules were administered 3 h before each session. Before and after SIT participants performed an incremental VO_2max_-test, a 30-min simulated cycling time-trial, as well as a 30-s cycling sprint test. Muscle biopsies were taken from *m. vastus lateralis*.

**Results:** SIT decreased the proportion of type IIx muscle fibers in all groups (*P* < 0.05). The relative number of type IIa fibers increased (*P* < 0.05) in HN (*P* < 0.05 vs. H), but not in the other groups. SIT had no significant effect on βhm. Compared with H, SIT tended to enhance 30-s sprint performance more in HN than in H (*P* = 0.085). VO_2max_ and 30-min time-trial performance increased in all groups to a similar extent.

**Conclusion:** SIT in hypoxia combined with nitrate supplementation increases the proportion of type IIa fibers in muscle, which may be associated with enhanced performance in short maximal exercise. Compared with normoxic training, hypoxic SIT does not alter βhm or endurance and sprinting exercise performance.

## Introduction

Interest in intermittent hypoxic training (IHT) to boost endurance and high-intensity exercise performance in athletes is growing (Hoppeler et al., 2008; McLean et al., 2014). This might partly be explained by the commercialization of user-friendly, normobaric hypoxicators to simulate altitude within the normal lowland habitat. Well-controlled studies have provided evidence that high-intensity hypoxic endurance training can enhance muscle mitochondrial and capillary density (Geiser et al., 2001; Vogt et al., 2001; Schmutz et al., 2010; Desplanches et al., 2014), as well as stimulate other markers of mitochondrial metabolism and biogenesis (Terrados et al., 1990; Melissa et al., 1997; Green et al., 1999; Zoll et al., 2006). IHT in the form of both endurance training (Vogt et al., 2001; Zoll et al., 2006) and sprint training (Faiss et al., 2013b; Puype et al., 2013) elevated muscle phosphofructokinase (PFK) mRNA and/or activity, as well as other markers of glycolytic metabolism and pH regulation. Nonetheless, research into the effects of IHT on sea-level exercise performance is equivocal, with the current literature suggesting higher training intensities involving anaerobic energy input to be more favorable than predominantly aerobic workouts (Hoppeler et al., 2008; Faiss et al., 2013a; McLean et al., 2014). This might be explained by impaired workload in endurance training due to inhibition of oxidative energy provision, resulting in higher glycolytic energy contribution and premature fatigue development (Weyand and Lee, 1999; Calbet et al., 2003; Wehrlin and Hallén, 2006).

Attention has recently shifted toward IHT in the form of sprint training (Faiss et al., 2013b, 2015; Galvin et al., 2013; Puype et al., 2013), because maximal power (Calbet et al., 2003) and anaerobic capacity (Friedmann et al., 2007) are well-maintained in hypoxia. This may allow for more explicit systemic and muscular adaptations due to elevated hypoxic and oxidative stress in conjunction with pertinent neuromuscular and neuromechanical loading (Morales-Alamo et al., 2012; McGinnis et al., 2014). Support for such a contention comes from recent studies showing that repeated sprint training in hypoxia (RSH), characterized by several short (< 30 s) sprints interspersed with incomplete recovery (exercise-to-rest ratio < 1:4), increased hypoxia-inducible factor-1α (HIF-1α) mRNA (Faiss et al., 2013b), and repeated-sprint ability (RSA) performance (Faiss et al., 2013b, 2015; Galvin et al., 2013) more than identical repeated sprint training in normoxia. However, the beneficial effect of RSH on exercise performance (Faiss et al., 2015) has been debated (Montero and Lundby, 2015), and needs to be confirmed. HIF-1α is implicated in the regulation of the genes controlling the expression of proteins involved in glycolysis and pH regulation (Porporato et al., 2011). In line with this, RSH has been shown to increase gene transcription of monocarboxylate transporter 4 (MCT-4) and carbonic anhydrase III (CA3) in muscle (Faiss et al., 2013b). We have shown that 6 weeks of sprint interval training (SIT), characterized by 30-s sprints interspersed with long recovery periods of 4–5 min, increases muscle MCT-1, but not MCT-4 protein content, irrespective of whether the training was performed in normoxia or in hypoxia (Puype et al., 2013). SIT in hypoxia but not in normoxia also elevated PFK activity (Puype et al., 2013), presumably due to increased glycolytic ATP turnover to compensate for impaired aerobic energy production during the hypoxic training workouts (Weyand and Lee, 1999; Calbet et al., 2003). Given that post-exercise phosphocreatine (PCr) resynthesis is impaired under hypoxic conditions (Haseler et al., 1999; Holliss et al., 2013; Vanhatalo et al., 2014), long recovery time between sprints is required to allow substantial recovery of PCr prior to each sprint (Bogdanis et al., 1996; Parolin et al., 1999) and may assist maintenance of high power output throughout the training session.

As the contribution of glycolysis to energy provision increases, buffering capacity becomes a pivotal determinant of the capacity to maintain high muscle power outputs. Counter-intuitively, the extent of H^+^ accumulation in muscle fibers during training does not seem to be the primary stimulus for the development of higher buffering capacity of homogenized muscle (βhm). Indeed, rat (Thomas et al., 2007) and human (Edge et al., 2006a) studies have shown that bicarbonate-induced myocellular alkalosis during work-matched interval training did not inhibit adaptations in βhm. Furthermore, a large acidic load (pH < 6.8) during training has been reported to reduce βhm, possibly due to cumulative transient decreases in βhm during consecutive training sessions (Bishop et al., 2008). In line with this rationale, performing SIT in hypoxic conditions might even impair βhm compared with similar training in normoxia. Alternatively, however, both “live high–train high” (Mizuno et al., 1990; Saltin et al., 1995) and “live high–train low” experiments (Gore et al., 2001) have shown that “live high” compared to “live low” enhances βhm. However, these findings have also been recently debated (Clark et al., 2004; Nordsborg et al., 2012), and the specific effect of IHT on βhm during “live low” conditions remains unclear.

Oral nitrate supplementation can enhance endurance exercise performance in hypoxia (Vanhatalo et al., 2011; Masschelein et al., 2012; Muggeridge et al., 2014), presumably by enhancing mitochondrial efficiency (Larsen et al., 2011) and/or by reducing the energy cost of muscle contraction (Bailey et al., 2010). It is well-documented that the fraction of aerobic energy provision gradually increases during intermittent sprints due to impaired re-activation of glycolysis (Parolin et al., 1999). Recent data indicate that nitrate intake increases blood flow and contractility to a greater extent in fast-glycolytic than in slow-oxidative whole muscle and muscle fibers (Hernández et al., 2012; Ferguson et al., 2013). This may explain the more explicit effects of nitrate supplementation on performance during high-intensity exercise requiring greater input of type II fibers for production of high power outputs at high contraction velocities (Vanhatalo et al., 2011; Breese et al., 2013; Bailey et al., 2015; Coggan et al., 2015). Higher muscle blood flow during recovery (Alvares et al., 2012) could conceivably facilitate the clearance of waste metabolites during intermittent maximal exercise bouts and could, amongst the other aforementioned mechanism, contribute to increased total work output during resistance training (Mosher et al., 2016). Furthermore, nitrate supplementation in hypoxia was shown to stimulate the rate of post-exercise muscle PCr resynthesis (Vanhatalo et al., 2011, 2014). Taken together, these results suggest that oral nitrate supplementation could enhance performance during SIT in hypoxia and by this means potentiate training adaptations.

We, therefore, aimed to investigate whether SIT performed in hypoxic conditions elicited greater muscular and performance adaptations compared to similar training performed in normoxic conditions. Secondly, we aimed to investigate whether oral nitrate supplementation during training enhanced the effects of SIT in hypoxia.

## Materials and methods

### Participants

Thirty healthy men were recruited from the student population at the KU Leuven by word of mouth and via announcements on social media. To avoid confounding effects due to prior altitude acclimatization, participants who were exposed to altitudes higher than 1500 m during the 6 months prior to the study were excluded from participation. From the initial sample of 30 eligible participants, one did not complete the study due to SIT intolerance, and two withdrew from the study for reasons unrelated to the study protocol. Twenty-seven participants completed the full study protocol and were included in the final data analyses (for general characteristics see Table 1). Participants were recreationally active [2.7 ± 1.6 h (*SD*) exercise participation per week; i.e., soccer, basketball, cycling, running, swimming, strength training], but had not engaged in a consistent training program or any sport at a competitive level. Participants were non-smokers and did not use medication or dietary supplements in the 3 months prior to the study or during the period of the study. They were instructed to maintain their habitual physical activity level and normal diet throughout the study. Participants received a summary table of nitrate-rich foods and were instructed to avoid these foods throughout the study period. The study was approved by the KU Leuven Biomedical Ethics Committee (B322201316517) and was conducted in accordance with the Declaration of Helsinki. All participants provided written informed consent after clearing medical screening and being fully informed about the content of the experiments and the risks involved.

**Table 1:** **Participant characteristics**.

	**N**	**H**	**HN**
Age (y)	23 ± 3	24 ± 2	25 ± 2
Height (cm)	180 ± 8	180 ± 6	182 ± 6
Body mass (kg)	74.0 ± 10.2	79.5 ± 12.1	78.5 ± 11.7

*Data are mean ± SD and represent baseline characteristics of the participants training in normoxia (F_i_O_2_ = 20.9%) while receiving placebo supplementation (N, n = 10), and participants training in hypoxia (F_i_O_2_ = 15.0%) while receiving either placebo (H, n = 8) or nitrate supplements (HN, n = 9)*.

### Study protocol

The study involved a test before (pretest) and after (posttest) a 5-week controlled SIT program. The pretest and posttest consisted of two experimental sessions separated by a 2-day interval. Between 3 and 2 weeks prior to the start of the study, participants completed two sessions of familiarization with the experimental procedures. In the first familiarization session, participants performed a maximal incremental VO_2max_-test on a cycle ergometer (Avantronic Cyclus II, Leipzig, Germany). The initial workload was set at 70 W and was increased by 30 W per min until volitional exhaustion. Respiratory gas exchange was measured continuously during the test (Cortex MetaLyzer II, Leipzig, Germany), and the highest oxygen uptake measured over a 30-s period was defined as the maximal oxygen uptake rate (VO_2max_). Participants then cycled for 15 min at 50 W to recover, after which a 30-s modified Wingate test (W_30s_) was performed. To avoid limitations of power output by co-ordination problems due to excessive cadence increase (cadence > 120 rev·min^−1^), cadence during W_30s_ was fixed at 100 rev·min^−1^ by using the isokinetic mode setting of the cycle ergometer. In the second familiarization session, participants completed a 30-min simulated time-trial (TT_30min_). They were instructed to keep their cadence between 80 and 100 rev·min^−1^, and adjust the resistance to develop the highest possible mean power output (W). Following familiarization, participants were matched into triplets by VO_2max_, mean power output during TT_30min_, mean power output during W_30s_, as well as body mass and height. Thereafter, the triplets were randomly assigned to one of three experimental groups. One group performed the SIT program in normoxia (F_i_O_2_ = 20.9%, *n* = 10) and received a placebo supplement (N). All other participants trained in hypoxia (F_i_O_2_ = 15.0%, ~2750 m), with eight participants receiving a placebo (H) and nine participants receiving a nitrate (HN, *n* = 9) supplement. Participants were not blinded for the normoxic vs. hypoxic training conditions. Supplements were ingested 3 h prior to each training session so as to produce high plasma nitrite levels during the training in HN(Webb et al., 2008). Nitrate was administered in the form of capsules containing 6.45 mmol NaNO_3_ (~400 mg molecular ${\text{NO}}_{3}^{-}$). Placebo capsules contained an equivalent amount of sodium (6.45 mmol) in the form of NaCl. All supplements were identical in appearance, and were administered single-blinded in N and double-blinded in H and HN.

### SIT training program

All SIT sessions were performed in the same normobaric hypoxic facility (SportingEdge, Sherfield on London, UK) set at either 20.9% F_i_O_2_ (N), or 15.0% F_*i*_O_2_ (~2750 m; H and HN). The ambient O_2_ fraction was checked before the start of each training session (${\text{MaxO}}_{2}^{+}$ A Scuba, Maxtec, Utah). Participants cycled on cycle ergometers (Avantronic Cyclus II, Leipzig, Germany) that were calibrated prior to the start of the study. Participants completed three training sessions per week, with each separated by 48-h of recovery. Each session consisted of intermittent 30-s maximal sprints, interspersed by 4.5 min active recovery intervals at 50 W. Cadence during the sprints was fixed at ~100 rev·min^−1^ by using the isokinetic operation mode of the ergometers. The number of sprints was increased from four in weeks 1–2, to five in weeks 3–4, and six in the final week. Including 5-min warm-up and cool-down @50 Watt, the training sessions lasted 30 min in week 1, increasing to 40 min in week 5. During each session participants were given verbal encouragement to perform maximally during each sprint. To evaluate the effect of SIT on arterial oxygenation, arterial oxygen saturation (SpO_2_) was monitored in the final week of the training period by pulse oximetry (Nellcor OxiMax N-600x, Mallinckrodt, St. Louis, MO) with a sensor placed 2 cm above the eyebrow.

### Pretest and posttest

All exercise testing was performed in normoxia. Participants were instructed to refrain from any strenuous physical activity for at least 48 h prior to the pretest. In order to minimize potential diet-induced variations in muscle metabolism, participants received a standardized carbohydrate-rich dinner (~1500 kcal; 65% carbohydrate, 15% fat, 20% protein) on the evening before each experimental day. For the first session they reported to the laboratory between 12:00 a.m. and 4:00 p.m. All participants received a standardized breakfast (~750 kcal, 70% carbohydrate, 10% fat, 20% protein) between 7:00 and 10:30 a.m. Participants completing sessions beyond 1:30 p.m. also received a standardized lunch (~650 kcal, 70% carbohydrate, 10% fat, 20% protein), with the last meal consistently being served between 3 and 2.5 h prior to the start of the experiments. Following a 1-h rest in a comfortable chair, a percutaneous needle biopsy (100–200 mg) was taken from the middle portion of the belly of the right *m. vastus lateralis* using a 5-mm Bergström-type needle under suction. Muscle samples were dissected in two parts. One part was rapidly frozen in liquid N_2_ and stored at −80°C until subsequent biochemical analyses. The other part was frozen in isopentane on liquid N_2_ and stored at −80°C for later histochemical analyses. Following the biopsy, participants warmed up for 20 min at incremental workloads corresponding to 70% (10 min) and then 90% (10 min) of their average power output recorded during the TT_30min_ familiarization session. During TT_30min_ heart rate was monitored continuously (Polar, Kempele, Finland) and blood lactate concentration was measured (Lactate Pro1, Arkray, Japan) at 10-min intervals from an earlobe capillary blood sample. Participants were allowed to drink water *ad libitum* and received on-line feedback about the time remaining to completion. No verbal encouragement was given. At the end of the experimental session, participants were instructed to refrain from any strenuous physical activity, before returning to the laboratory for the second session 2 days later. For this second session they arrived between 6 a.m. and 11 a.m. after an overnight fast. Following a 20-min rest period, they performed a maximal incremental VO_2max_-test on the cycle ergometer (Avantronic Cyclus II, Leipzig, Germany). Initial workload was set at 70 W and was increased by 30 W every 3 min until volitional exhaustion. Thereafter participants cycled for 15 min at 50 W to recover, whereupon the W_30s_ commenced. The cycle ergometer was set in the isokinetic mode with cadence fixed at 100 rev·min^−1^. During both tests heart rate was monitored continuously (Polar, Kempele, Finland). Standardized verbal encouragement was given only during W_30s_. Respiratory gas exchange was continuously measured (Cortex MetaLyzer II, Leipzig, Germany) during the incremental test, VO_2max_ was determined as the highest oxygen uptake rate measured over a 30-s period. Maximal power output (MPO) was calculated by summing the workload during the last full stage, plus 30 W multiplied by the fraction of the final stage completed. Capillary blood samples for lactate determination (Lactate Pro1, Arkray, Japan) were taken from the earlobe at the end of each workload during the incremental test, and power outputs corresponding to 2 and 4 mmol·L^−1^ blood lactate levels were extrapolated on the lactate-power curve. Blood lactate was also determined 1, 2, and 3 min after the W_30s_. Room temperature (18–20°C), oxygen content (20.9%), air humidity (40%) as well as air ventilation were standardized. Pretests and the posttests were performed on the same days of the week and time of the day within each participant. The posttest commenced 3 or 4 days following the last training session to eliminate acute physiological effects due to the prior training session.

### Analysis of muscle samples

#### Citrate synthase activity

Enzymatic activity of citrate synthase (CS) was measured by standard colorimetric method. Briefly, 5 mg of wet muscle tissue was dissolved in 400 μL of ice-cold homogenization buffer (5 mM Hepes, 1 mM EGTA, 0.1% Triton X-100, 1 mM Dithiothreitol, pH 8.7). Protein concentration was determined with a DC protein assay (Bio-Rad). After dilution to 0.5 μg·μL^−1^, samples and standards were loaded on a 96 well plate to perform the assay in triplicate. CS catalyzes the reaction between acetyl coenzyme A and oxaloacetic acid resulting in citric acid and CoA with a thiol group (CoA-SH). Measurement of its activity is based on the binding of CoA-SH to 5,5′-dithio-bis-(2-nitrobenzoic acid) (DTNB) to form 2-nitro-5-thiobenzoate (TNB). The spectrophotometric absorbance intensity of TNB was measured at 412 nm and CS activity was calculated and expressed as μmol·min^−1^·g^−1^. The average coefficient of variation (CV) as determined from the triplicate measures was 4.4%.

#### Buffering capacity of homogenized muscle (βhm)

Details of the titration method for analysis of βhm have been described elsewhere (Edge et al., 2006b). Briefly, freeze-dried muscle samples (1.7–2.5 mg dm) were dissected from blood and connective tissue and homogenized on ice in a sodium fluoride containing buffer (33.3 μl 10 mM NaF per mg dm). The homogenates were warmed in a hot water bath at 37.4°C for 5 min. Basal pH measurement was performed with a glass microelectrode (MI-410, Microelectrodes, Bedford, NH, USA) connected to a pH meter (Lab 850, Schott Instruments GmbH, Mainz, Germany). The homogenates were first adjusted to pH ~7.2 with sodium hydroxide (0.02 M NaOH). Then a serial addition of 2 μL hydrochloric acid (0.01 M HCl) was titrated until a pH of ~6.1 was reached. After each titration, the homogenates were briefly vortexed to ensure a homogeneously mixed solution. The number of moles of H^+^ per kg dry muscle required to change pH from 7.1 to 6.5 was interpolated from the fitted titration trend line and expressed as mmol H^+^ per kg dm per unit pH as a unit for βhm. Each sample was measured in duplicate from which the mean was taken. The average CV as determined from the duplicate measures was 5.1%.

#### Muscle carnosine concentration

Details of muscle carnosine concentration determination by high performance liquid chromatography (HPLC) have been described elsewhere (Mora et al., 2007). Briefly, about 5 mg of dry muscle was dissected from blood and connective tissue and extracted in a buffer containing perchloric acid (0.5 M PCA) and 1 mM EDTA. After centrifuging the samples for 4 min at 13,000 rev·min^−1^ at 4°C, the supernatant was collected and neutralized with a potassium bicarbonate containing buffer (2.1 M KHCO). Samples were placed on ice for 5 min to allow CO_2_ to escape, after which they were centrifuged at 5000 rev·min^−1^ for 4 min at 4°C. The supernatant was then filtered through a 0.22 μm membrane filter where after 20 μL of supernatant was injected into a Perkin-Elmer HPLC system with an Atlantis HILIC Silica column (4.6 × 150 mm, 3 μm). Mobile phase A contained 0.65 mM ammonium acetate in ultrapure water/acetonitrile (25:75 ratio) at a pH of 5.5. Mobile phase B contained 4.55 mM ammonium acetate in ultrapure water/acetonitrile (70:30 ratio) at a pH of 5.5. A linear gradient from 100% phase A to 100% phase B in 13 min at a flow rate of 1.4 mL·min^−1^ was used for separation. Separation was monitored at a wavelength of 214 nm with a UV detector. The average CV as calculated from 12 duplicate injections in the HPLC system was 1.5%.

#### Muscle fiber type composition

Serial 7-μm-thick cryosections were cut with a cryostat at −20°C. Cryosections were blocked for 60 min in phosphate buffered saline (PBS) containing 1% BSA. Hereafter they were incubated in primary antibodies for myosin heavy chain I (MHCI) (BA-F8, Developmental Studies Hybridoma Bank) and MHCIIa (SC-71, Developmental Studies Hybridoma Bank) dissolved in PBS with 0.5% BSA for 120 min. Dilutions of primary antibodies for MHCI and MHCII were 1:50 and 1:100, respectively. After washing in PBS, cryosections were incubated in appropriate conjugated secondary antibodies (type I: Alexa 647 goat anti-mouse IgG2b, Invitrogen, diluted 1:300 in PBS with 0.5% BSA; type IIa: Alexa 350 goat anti-mouse IgG1, Invitrogen, diluted 1:300 in PBS with 0.5% BSA) for 60 min. Additionally, together with the secondary antibodies, membranes were stained using wheat germ agglutinin (WGA) Texas Red (Life Technologies). Slides were visualized by fluorescence microscopy (Nikon E1000, Nikon, Boerhavedorp, Germany). The epifluorescence signal was recorded using Cy5, DAPI, and Texas Red excitation filters for visualization of type I fibers, type IIa fibers, and cell membranes, respectively. Muscle fibers were classified as type I, type IIa, or type IIx (unstained fibers). Photos of the slides were analyzed with ImageJ software (version 1.41, National Institutes of Health, USA). Only fibers with adequate cross-sections showing no signs of distortion or folding were counted. 225 ± 27 (*SD*) fibers were analyzed per biopsy.

### Statistical analysis

Differences in baseline values between N and H and between H and HN were tested using a Student's *t*-test. Main and interaction effects were evaluated by two-way (group × time) repeated measures ANOVA (SigmaStat and SigmaPlot software, Chicago, IL, USA). We performed two separate ANOVA's to test the two *a priori* hypotheses: N was compared with H to evaluate whether SIT yielded different effects in hypoxia vs. normoxia; H was compared with HN to evaluate whether nitrate administration was able to potentiate the effects of training in hypoxia. Tukey's honestly significant difference *post hoc*-test was run whenever appropriate to identify specific effects. A probability level *P* < 0.05 was considered statistically significant. All data are expressed as mean ± standard error of the mean (*SEM*) unless otherwise stated.

## Results

### Arterial O_2_-saturation during training (Figure 1)

Arterial O_2_-saturation (SpO_2_) was continuously measured during the SIT sessions in week 5. In N, resting SpO_2_-values were 98.7 ± 0.4 and 97.6 ± 0.5% at the start and at the end of the sessions (n.s.). Corresponding values in H were lower, both at the start (91.0 ± 0.8%) and at the end of the sessions (89.3 ± 1.4%; *P* < 0.05). Compared with sprint 1, post-exercise SpO_2_-values were lower in the latter sprints of the session in both groups. Each sprint also reduced SpO_2_ more in H (–6.0 ± 0.6%) than in N (−1.9 ± 0.2%, *P* < 0.05). SpO_2_-values were not significantly different between H and HN: average SpO_2_ during SIT was 85.0 ± 0.6% in H vs. 86.7 ± 0.3% in HN (*P* = 0.20).

**Figure 1:**
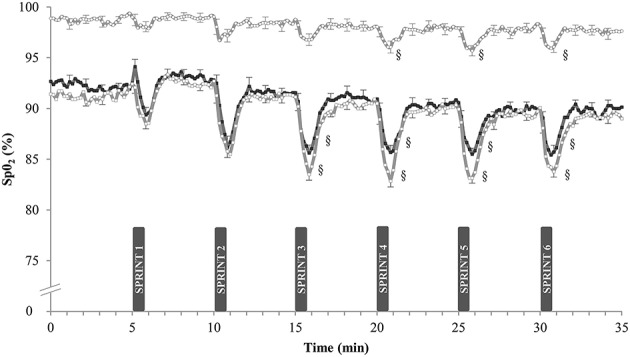
**Effect of SIT on arterial oxygen saturation**. Data are mean ± SEM and represent arterial oxygen saturation (%SpO_2_) during the final week of SIT. Participants performed six 30-s all-out sprints interspersed by 4 min and 30 s of active recovery on a cycle ergometer. One group trained in normoxia (F_i_O_2_ = 20.9%) while receiving placebo (N, ◦). The other groups trained in hypoxia (F_i_O_2_ = 15.0%) while receiving either placebo (H, □) or nitrate supplements (HN, ■). §, *P* < 0.05 compared to SPRINT 1.

### Training performance (Figure 2)

**Figure 2:**
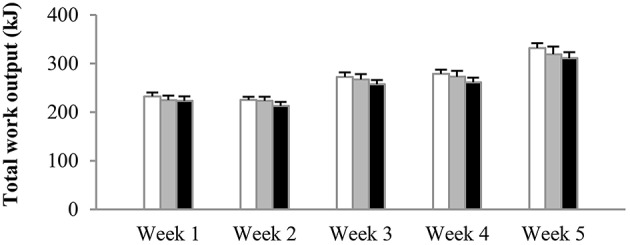
**Effect of hypoxia and nitrate intake on training workload**. Data are mean ± SEM and represent average total work output (kJ) per training session per week. The number of sprints per session was increased from four in weeks 1–2, to five in weeks 3–4, and six in the final week. One group trained in normoxia (F_i_O_2_ = 20.9%) while receiving placebo (N, open bars). The other groups trained in hypoxia (F_i_O_2_ = 15.0%) while receiving either placebo (H, gray bars) or nitrate supplements (HN, black bars).

The number of sprints per session was increased from four in weeks 1–2, to five in weeks 3–4, and to six in week 5. Irrespective of the experimental group, mean power output in sprint 1 on average was 667 ± 31 W, decreasing to 561 ± 25 W in the final sprint of the session. There were no significant differences in power output between experimental groups at any time during training. Average total work done (kJ) per training session per week was similar between the groups from the start to the end of the training period (Figure 2). Accordingly, total work output over the 5-week training period was 1340 ± 39 kJ in N, 1311 ± 52 kJ in H, and 1261 ± 51 kJ in HN (*P* > 0.50). Blood lactate concentrations at the end of the training sessions peaked at ~14–15 mmol·L^−1^ on average in all groups.

### Exercise performance (Table 2)

Baseline values of the incremental exercise test, TT_30min_ and W_30s_ were not significantly different between N and H (*P* > 0.05) or H and HN (*P* > 0.05). Compared with the pretest, VO_2max_ in the posttest was increased (*P* < 0.05) by ~16% in N vs. ~11% in both H and HN (*P* < 0.05), but there were no significant differences in VO_2max_ or change in VO_2max_ between N and H (*P* = 0.26) or between H and HN. Similarly, SIT increased (*P* < 0.05) time to exhaustion (10–11%), peak power output (8–10%), and power output corresponding to 4 mmol·L^−1^ blood lactate concentrations (5–11%) in the three experimental groups without significant differences between N and H or H and HN. Mean power output during the TT_30min_ in the pretest was, on average, ~200 W. Training tended to increase mean power output during the TT_30min_ in N (+4%, *P* = 0.062) and significantly increased power output during the TT_30min_ in H and HN (+8%, *P* < 0.05), but there were no significant differences between N and H or H and HN. Blood lactate concentrations during the TT_30min_ were, on average, ~6 mmol·L^−1^ at a heart rate of ~174 b·min^−1^ in each group in both the pretest and the posttest (data not shown). Mean power output during W_30s_ in the pretest also was similar between the groups (660–670 W). SIT increased power output by ~6% in N and H (*P* < 0.05), vs. +12% in HN (*P* < 0.05), yet differences between H and HN were not significant (HN, *P* = 0.085). Accordingly, W_30s_ produced similar peak blood lactate concentrations (~10–12 mmol·L^−1^) in the three experimental groups in both the pretest and the posttest.

**Table 2:** **Effects of training and nitrate supplementation on physiological parameters and exercise performances**.

	**N**		**H**		**HN**	
	**Pretest**	**Posttest**	**Pretest**	**Posttest**	**Pretest**	**Posttest**
**INCREMENTAL VO**_2max_ **TEST**						
VO_2max_ (mL·min^−1^·kg^−1^)	53.5 ± 2.6	62.3 ± 3.4^*^	54.3 ± 4.9	60.2 ± 3.5^*^	51.2 ± 2.2	56.9 ± 2.3^*^
Time to exhaustion (min)	23.1 ± 1.1	25.5 ± 1.0^*^	23.8 ± 1.8	26.4 ± 1.7^*^	22.4 ± 1.1	25.0 ± 1.2^*^
Peak power (W)	275 ± 12	295 ± 11^*^	278 ± 18	304 ± 17^*^	264 ± 11	290 ± 12^*^
Peak heart rate (beats·min^−1^)	188 ± 3	186 ± 3	188 ± 2	188 ± 3	190 ± 4	191 ± 3
Peak blood lactate (mmol·L^−1^)	11.2 ± 0.7	11.4 ± 0.4	11.0 ± 1.1	11.8 ± 1.0	12.1 ± 0.9	12.5 ± 0.6
Power output at 2 mmol·L^−1^ (W)	175 ± 10	189 ± 13^#^	161 ± 20	179 ± 14^*^	148 ± 14.1	172 ± 11^*^
Power output at 4 mmol·L^−1^ (W)	216 ± 10	227 ± 13^*^	213 ± 18	230 ± 14^*^	196 ± 12.0	217 ± 11^*^
**TT_30min_**						
Mean power output (W)	203 ± 10	211 ± 12^#^	205 ± 16	221 ± 15^*^	193 ± 13	209 ± 12^*^
**W_30s_**						
Mean power output (W)	662 ± 23	699 ± 21^*^	677 ± 25	719 ± 34^*^	663 ± 45	746 ± 41^*^
Peak blood lactate (mmol·L^−1^)	9.2 ± 0.7	11.1 ± 0.9^*^	11.5 ± 0.7	12.4 ± 0.5	9.8 ± 0.7	12.4 ± 0.4^*^

*Data are mean ± SEM for the maximal incremental VO_2max_-test, the 30-min simulated time-trial (TT_30min_), and the 30-s Wingate test (W_30s_) in the pretest and in the posttest. One group trained in normoxia (F_i_O_2_ = 20.9%) while receiving placebo (N, n = 10). The two other groups trained in hypoxia (F_i_O_2_ = 15.0%) and received either placebo (H, n = 8) or nitrate supplements (HN, n = 9)*.

* *P < 0.05 compared to the pretest*.

# *P < 0.10 compared to the pretest*.

### Muscle fiber type composition (Table 3)

The relative number of type I (~45–50%), type IIa (~40–45%), and type IIx fibers (~10%) in *m. vastus lateralis* in the pretest was similar between N and H (*P* > 0.05 for all fiber types) as well as between H and HN (*P* > 0.05 for all fiber types). SIT reduced the proportion of type IIx fibers in all the groups (*P* < 0.05). In the posttest the proportion of type IIa fibers was higher in HN than in H (*P* < 0.05), with training significantly increasing the proportion of IIa fibers from 45 to 56% in HN (*P* < 0.05), but not in N (main effect of time *P* = 0.07) or H (*P* = 0.40). Similar changes occurred for fiber-specific CSAs, which also were similar between N and H or H and HN in the pretest (*P* > 0.05 for all fiber types). SIT reduced the relative CSA of type IIx fibers in all the groups (*P* < 0.05). Conversely, type IIa relative CSA increased in HN (+11%, *P* < 0.05) only and was significantly greater in HN compared to H in the posttest (*P* < 0.05). SIT did not alter mean fiber CSA (μm^2^) of the different fiber types, except for type IIx mean fiber CSA in H which increased from the pretest to the posttest (*P* < 0.05). However, type IIx fibers were observed in only half of the participants in H (*n* = 4), in which only few type IIx fibers were observed in the pretest [44 ± 33(*SD*)] and in the posttest [30 ± 28 (*SD*)]. Hence interpretation should be performed with caution. Mean fiber-specific CSAs were not significantly different between N and H or H and HN at any time.

**Table 3:** **Effects of training and nitrate intake on muscle fiber composition**.

	**N**		**H**		**HN**	
	**Pretest**	**Posttest**	**Pretest**	**Posttest**	**Pretest**	**Posttest**
**RELATIVE FIBER NUMBER (%)**						
Type I	49 ± 3	49 ± 3	51 ± 5	53 ± 5	45 ± 4	39 ± 2
Type IIa	42 ± 3	49 ± 3	42 ± 4	44 ± 4	45 ± 2	56 ± 2^*^^,^^†^
Type IIx	9 ± 2	2 ± 1^*^	7 ± 2	3 ± 2^*^	10 ± 2	5 ± 1^*^
**RELATIVE FIBER CSA (%)**						
Type I	48 ± 3	51 ± 2	48 ± 5	50 ± 5	44 ± 4	38 ± 2
Type IIa	44 ± 3	47 ± 2	45 ± 5	47 ± 4	47 ± 2	58 ± 2^*^
Type IIx	8 ± 2	2 ± 1^*^	7 ± 2	3 ± 1^*^	8 ± 2	4 ± 1^*^
**FIBER CSA (μm^2^)**						
Type I	4769 ± 604	5214 ± 501	5269 ± 204	5843 ± 462	4998 ± 402	5106 ± 434
Type IIa	4952 ± 429	4769 ± 411	5978 ± 281	6554 ± 348	5365 ± 534	5497 ± 573
Type IIx	4507 ± 444	4302 ± 428	4929 ± 664	6078 ± 474^*^	3976 ± 405	4438 ± 537

*Data are mean ± SEM for muscle fiber cross-sectional area (CSA) and fiber number in m. vastus lateralis in the pretest and in the posttest. One group trained in normoxia (F_i_O_2_ = 20.9%) while receiving placebo (N, n = 10). The other groups trained in hypoxia (F_i_O_2_ = 15.0%) and received either placebo (H, n = 8) or nitrate supplements (HN, n = 9)*.

* *P < 0.05 compared to pretest*.

† *P < 0.05 group × time interaction*.

### Muscle biochemistry (Table 4)

Baseline values for muscular buffering capacity, carnosine content, and maximal citrate synthase activity measured in homogenized muscle tissue were not significantly different between N and H or H and HN (*P* > 0.05). Buffering capacity of homogenized muscle was unaffected by SIT irrespective of the experimental condition. SIT on average increased muscle carnosine content by ~13% in H and HN (main effect of time *P* < 0.05, no significant *post hoc* time effects within H (*P* = 0.11) or HN (*P* = 0.13)), but not in N (+6%, *P* = 0.072). Nonetheless, the increase in carnosine content was not significantly different between N and H (*P* = 0.48), and there were no significant differences in muscle carnosine between the groups in either the pretest or the posttest. Maximal citrate synthase activity increased by 54% in N (*P* < 0.05) and by just under half that in H (22%) and HN (25%) (*P* < 0.05), yet changes were not significantly different between N and H (*P* = 0.10).

**Table 4:** **Effects of training and nitrate intake on biochemical measurements in muscle**.

	**N**		**H**		**HN**	
	**Pretest**	**Posttest**	**Pretest**	**Posttest**	**Pretest**	**Posttest**
βhm (mmol H^+^·kg dm^−1^·pH^−1^)	136 ± 6	136 ± 6	138 ± 5	137 ± 6	125 ± 6	128 ± 6
Carnosine content (mmol·kg dm^−1^)	33.3 ± 2.3	35.3 ± 2.2	33.8 ± 2.9	38.2 ± 2.9	30.2 ± 2.2	34.0 ± 2.6
Citrate synthase activity (μmol·min^−1^·g^−1^)	190 ± 19	292 ± 24^*^	231 ± 10	282 ± 16^*^	216 ± 17	269 ± 8.2^*^

*Data are mean ± SEM for buffering capacity of homogenized muscle (βhm), carnosine content, and citrate synthase activity in the pretest and the posttest. One group trained in normoxia (F_i_O_2_ = 20.9%) while receiving placebo (N, n = 10). The other groups trained in hypoxia (F_i_O_2_ = 15.0%) and received either placebo (H, n = 8) or nitrate supplements (HN, n = 9)*.

* *P < 0.05 compared to pretest*.

## Discussion

The most striking results from the current study are that (a) 5 weeks of SIT, performed in hypoxic conditions, significantly increased the fraction of type IIa muscle fibers, but only when completed with concomitant dietary nitrate supplementation and (b) SIT performed in hypoxic conditions does not ameliorate physiological adaptations yielding enhanced aerobic or anaerobic endurance exercise performance.

We administered 400 mg of molecular nitrate ~3 h before each SIT session in HN, which has previously been shown to significantly increase the plasma nitrite concentration (Wylie et al., 2013) and improve oxygen-efficiency of the skeletal muscle during exercise (Bailey et al., 2010; Larsen et al., 2011). We postulated that this might delay fatigue development and thereby attenuate the reduced training intensity often shown during hypoxic sprint training (Kelly et al., 2014; Thompson et al., 2015). Training workloads were, however, similar between all groups, regardless of whether the training was performed in normoxia (N) or in hypoxia, either with (HN) or without (H) nitrate supplementation. Thus, the degree of neuromechanical activation during training was similar between all experimental conditions.

Even in the absence of fiber hypertrophy, fiber type transition from IIx to IIa might be expected during short-term SIT involving 30-s sprints in healthy volunteers (for review, see Ross and Leveritt, 2001). In keeping with the published findings (Allemeier et al., 1994), 5 weeks of SIT did not induce muscle fiber hypertrophy in the current study. Consistent with this, SIT in normoxia reduced the relative type IIx fiber number, whilst the proportion of type IIa fibers tended to increase. SIT in hypoxia (H) also reduced type IIx fiber number, but did not alter the fraction of type IIa fibers. Interestingly, when nitrate supplementation was provided during SIT in hypoxic conditions (HN), relative type IIa fiber number increased from 45 to 56%.

Previous evidence indicates that nitric oxide (NO) plays a pivotal role in MHC-based muscle fiber type transition (Smith et al., 2002; Martins et al., 2012; Suwa et al., 2015). NO is suggested to increase inhibitory phosphorylation of glycogen synthase kinase (GSK)-3β in rat fast-twitch muscle, promoting nuclear factor of activated T-cell c1 (NFATc1) dephosphorylation and nuclear accumulation, resulting in a fast-to-slow fiber type transition (Martins et al., 2012). Furthermore, pharmacological inhibition of NO-synthase (NOS) activity by N^G^-nitro-L-arginine-methyl-ester (L-NAME) negated overload-induced type II to I fiber type transition (Smith et al., 2002). Results from both rodent (Hernández et al., 2012; Ferguson et al., 2013) and human (Bailey et al., 2015; Coggan et al., 2015) exercise studies indicated that oral nitrate intake exerts its actions primarily in type II muscle fibers by increasing blood flow (Ferguson et al., 2013), by elevating sarcoplasmic reticulum calcium stores and the expression of calcium handling proteins such as calsequestrin 1 and the dihydropyridine receptor (Hernández et al., 2012), as well as by reducing muscle metabolic perturbation (Vanhatalo et al., 2011). Nitrate supplementation also stimulated rate of force development (Hernández et al., 2012) and power output (Bailey et al., 2015; Coggan et al., 2015) during high-intensity and high-velocity muscle contractions. Thus, while endogenous NO production during acute exposure of lowlanders to hypoxia is reduced, due to inhibition of the L-arginine–NOS pathway (Lundberg et al., 2008), exogenous nitrate provides an alternative pathway to stimulate NO production via nitrate to nitrite to NO conversion (Lundberg et al., 2008). Given that NO probably plays an important role in exercise-induced MHC-based adult fiber type transitions, it is plausible that oral nitrate intake during SIT in hypoxia served as an adequate back-up mechanism for NO-induced muscle fiber transformation to compensate for impaired NOS activity. However, in contrast to what could be expected from the current literature, i.e., stimulation of type IIx to IIa and IIa to type I fiber type transition (Smith et al., 2002; Martins et al., 2012; Suwa et al., 2015), nitrate supplementation during hypoxic SIT did not stimulate the transition of type IIa to type I muscle fibers. Follow-up studies are needed to elucidate the cellular mechanisms by which oral nitrate intake can stimulate the conversion to type IIa muscle fibers during SIT in hypoxia.

Despite a decrease in the fraction of IIx muscle fibers in all conditions and an increase in the fraction of IIa muscle fibers in HN, there was no change in βhm with training in any of the three groups. We postulated that SIT in hypoxia, due to an enhanced contribution of glycolysis to ATP production, might impair βhm compared with identical training in normoxia. Contrary to our hypothesis, however, SIT did not alter βhm, regardless of whether the training was completed in normoxia or hypoxia. Our data shows that SIT is not an adequate strategy to improve βhm, at least in healthy recreationally active volunteers during short-term training. Findings from the present and earlier studies support the opinion that high-intensity interval training (HIT) at workloads corresponding to 120–170% of the lactate threshold (Edge et al., 2006a,b) is probably more effective to raise βhm than explicit “glycolytic training” via SIT (Nevill et al., 1989; Harmer et al., 2000; Baguet et al., 2011). Adaptations of βhm during long-term SIT in elite sprinters may be different, given their substantially higher proportion of type II fibers and higher glycolytic capacity.

The physicochemical buffer capacity of muscle (~βhm) is composed of proteins, inorganic phosphate, bicarbonate, and the histidine-containing dipeptide carnosine. The histidine containing dipeptide content of muscle (carnosine in human muscle) contributes ~7–8% to total βhm (Harris et al., 1990; Mannion et al., 1992; Hill et al., 2007), and elevations through training or dietary supplementation remain a plausible mechanism for increasing βhm. Accordingly, we determined the effect of SIT on muscle carnosine content and observed no significant effect in normoxic conditions. However, a main effect of time (pretest vs. posttest), though without significant *post hoc* effects, was found for the increase in muscle carnosine content following training in hypoxic conditions (H and HN), yet the training-induced changes were not significantly different between N and H. Earlier experiments in our laboratory (Puype et al., 2013) have shown that SIT in hypoxia did not increase muscle carnosine content (unpublished observations), suggesting some equivocality in the findings across our own studies. This would seem in line with the existing literature, given that SIT has previously been shown to substantially increase muscle carnosine content in one study (Suzuki et al., 2004), although no such training-effect has been shown in any other longitudinal intervention study using SIT (Baguet et al., 2011) or resistance training involving either short (~10 s) (Kendrick et al., 2009) or longer (~20–45 s) series of maximal muscle contractions in normoxia (Mannion et al., 1994). Future studies performing single-fiber determination of muscle carnosine are warranted for clear interpretation of training-induced changes in fiber-specific myocellular carnosine content.

Buffering capacity of homogenized muscle, as measured in the present study, is different to muscle buffering capacity *in vivo* and reflects the altered state and chemistry of muscle after homogenization. Assuming that there would be no change in any other source of myocellular buffering, it could be estimated that a 13% increase in muscle carnosine content (i.e., 4 mmol·kg dm^−1^) would increase homogenized muscle buffering capacity over the titration pH range of 7.1 to 6.5 by 2.21 mmol H^+^·kg dm^−1^·pH^−1^, given that at pH 7.1 and pH 6.5 34.9 and 68.1% of the additional carnosine content is already in the protonated form, respectively (Harris et al., 1990). This represents an increase of ~1.7% of the initial βhm in the present study and falls below the detection limit of the titration assay used. However, as we don't know what muscle buffering capacity *in vivo* is over this range, we cannot calculate the importance of this increase.

We also evaluated the effect of SIT on muscle oxidative capacity by measuring citrate synthase (CS) maximal activity. SIT substantially elevated CS activity (+25–50%), independent of whether the training was performed in normoxia or in hypoxia. These findings are in contrast with reports from single-leg studies reporting greater increases in CS activity following endurance training (30 min at 65–75% maximal work capacity) in hypoxic conditions compared to training at similar absolute workloads in normoxic conditions (Terrados et al., 1990; Melissa et al., 1997; Green et al., 1999). However, if the training workloads are matched for relative intensity, endurance training in hypoxic conditions abolishes rather than augments training-induced changes in muscle oxidative function (Bakkman et al., 2007). Training workloads during SIT in the present study were similar between normoxic and hypoxic conditions in both absolute and relative terms. In line with previous reports, SIT in normoxic conditions increased CS activity (for review see Sloth et al., 2013), but sprint training in hypoxic conditions did not augment adaptations in muscle oxidative capacity (Faiss et al., 2013a; Puype et al., 2013).

The hypothesis driving the current study was underpinned by the notion that performing SIT in hypoxic conditions, alone or in combination with oral nitrate intake, might yield specific physiological adaptations to boost exercise performance. Anaerobic glycolysis accounts for about 50% of the total ATP production during a 30-s maximal exercise bout (Putman et al., 1995). It is therefore reasonable to postulate that a higher proportion of type IIa muscle fibers, providing a higher capacity for glycolytic ATP production, should be ergogenic during a 30-s all-out exercise. As discussed above, hypoxic SIT in conjunction with oral nitrate supplementation increased relative type IIa muscle area compared with hypoxic SIT alone. Interestingly, this also tended to translate into a greater gain in power output during W_30s_ (+12% in HN vs. +6% in H, *P* = 0.08), which indicates that short-term oral nitrate supplementation in conjunction with SIT may be a valid strategy to enhance performance in “glycolytic” exercise events such as a 400-meter dash, by contributing to a beneficial fiber type shift. Future studies should seek to confirm this possibility. It is also important to emphasize that we did not study the effect of nitrate intake during SIT in normoxia, neither did we study exercise performance in hypoxia. Thus, we cannot extrapolate our findings to these conditions. Contrary to 30-s sprint performance, determinants of aerobic exercise capacity were similar between the groups. Five weeks of SIT enhanced cycling power output corresponding to 2 and 4 mmol·L^−1^ blood lactate levels, VO_2max_, and 30-min cycling time-trial performance, independent of whether the training was performed in normoxia, or in hypoxia with or without oral nitrate supplementation. These findings indicate that short-term SIT in hypoxic compared to normoxic conditions is not an advantageous strategy for enhancing normoxic endurance exercise performance in recreationally active individuals.

In conclusion, the current experiment demonstrated that oral nitrate supplementation during short-term sprint-interval training increased the proportion of type IIa muscle fibers in muscle, which may contribute to enhanced performance in short maximal exercise events requiring a very high glycolytic rate. Compared with SIT in normoxia, SIT in hypoxia did not generate beneficial physiological adaptations yielding enhanced aerobic or anaerobic endurance exercise performance.

## Author contributions

Conception and design of the study: PH and SD. All authors contributed to the collection and interpretation of the data, and reviewed and approved the final manuscript written by PH and SD.

### Conflict of interest statement

The authors declare that the research was conducted in the absence of any commercial or financial relationships that could be construed as a potential conflict of interest.
